# The effects of recombinant human activated factor VII and tranexamic acid on traumatic bleeding and mortality in mice

**DOI:** 10.1016/j.rpth.2026.103436

**Published:** 2026-03-26

**Authors:** Bilgimol Chumappumkal Joseph, Juan Andres De Pablo-Moreno, Nicca Falah, Abraham Wentzel, Mia Lora Cacho, Eduardo Frias-Anaya, Miguel A. Lopez-Ramirez, Annette von Drygalski

**Affiliations:** 1 Division of Hematology and Cellular Therapy, Department of Medicine, University of California San Diego, La Jolla, California, USA; 2 Department of Veterinary Medicine, School of Biomedical and Health Sciences, Universidad Europea de Madrid, Villaviciosa de Odon, Spain; 3 Department of Physiology, School of Veterinary Medicine, Complutense University of Madrid, Madrid, Spain

**Keywords:** bleeding, blood loss, coagulopathy, rhFVIIa, surgery, tranexamic acid, trauma

## Abstract

**Background:**

Tranexamic acid (TXA) and recombinant human activated factor (F)VII (rhFVIIa) both control severe traumatic bleeding, with mortality benefit only demonstrated for TXA.

**Objectives:**

To compare the effects of rhFVIIa and TXA on bleeding, trauma-induced coagulopathy (TIC), inflammation, and survival in a murine trauma model.

**Methods:**

Liver laceration (LL) was employed to induce TIC. C57BL/6J mice were pretreated intravenously with saline, rhFVIIa (3 mg/kg), or TXA (10 mg/kg). Blood loss, coagulopathy (activated partial thromboplastin time [aPTT], FII, FV, FVIII, FX, thrombin-antithrombin [TAT], and fibrinogen), and fibrinolysis (tissue-type plasminogen activator, plasmin-α2-antiplasmin complexes, and D-dimer) were analyzed 60 minutes after LL. Cytokines were measured at 60 minutes and 6 hours. Pulmonary fibrin deposition and survival were evaluated for up to 7 days.

**Results:**

Both rhFVIIa and TXA reduced blood loss compared with saline-treated mice after LL. Saline-treated mice developed TIC (prolonged aPTT, increased TAT complex formation, and selective depletion of FV, FVIII, and fibrinogen). rhFVIIa overcorrected the aPTT and increased TAT complex levels, whereas TXA normalized these parameters. Both agents reduced tissue-type plasminogen activator and plasmin-α2-antiplasmin complex formation; however, rhFVIIa failed to suppress D-dimer formation and exacerbated interleukin-6 formation. Pulmonary fibrin deposition and microthrombi occurred exclusively in rhFVIIa-treated mice (days 1, 2, and 7), accompanied by reduced survival (∼50%) compared with TXA (∼80%).

**Conclusion:**

While both agents reduced bleeding, rhFVIIa promoted prothrombotic and inflammatory responses, which were associated with increased mortality. Our findings highlight the unmet need for targeted interventions to reduce TIC while minimizing thromboinflammatory risk.

## Essentials

• Severe trauma disrupts hemostasis and can result in exsanguination if left untreated.
• We used a murine liver laceration model to study rhFVII and TXA.
• Both treatments reduced bleeding, but rhFVIIa decreased survival.
• rhFVIIa increased thromboinflammation, highlighting the need for novel therapies.

## Graphical abstract

**Figure:**
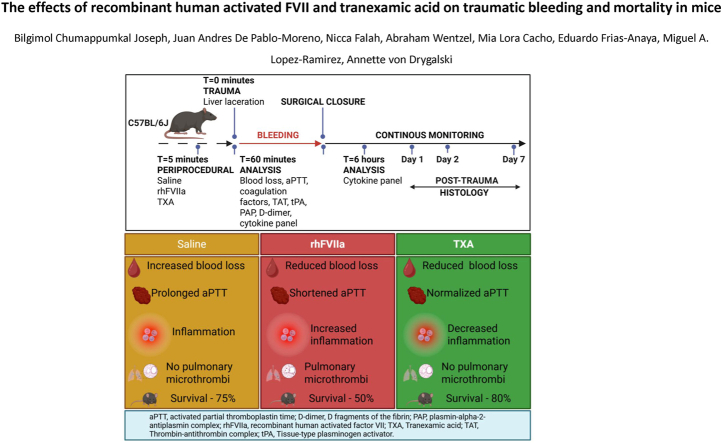

## 1 Introduction

Uncontrolled hemorrhage remains a leading cause of early mortality following trauma, accounting for nearly 40% of trauma-related deaths worldwide [[1], [2], [3]]. Despite advances in surgical techniques and critical care, trauma-induced coagulopathy (TIC) continues to complicate management and is associated with worsened outcomes [4,5]. TIC is a complex and dynamic condition characterized by impaired thrombin generation [6], selective depletion of coagulation factor (F)V and FVIII [7], platelet dysfunction [8], and excessive fibrinolysis [9]. Identifying therapeutic strategies that effectively mitigate hemorrhage and stabilize the hemostatic system without provoking thrombotic complications remains an unmet clinical need.

Recombinant human activated FVII (rhFVIIa) was originally developed as a bypassing agent for the treatment of hemophilia with inhibitors [10]. Since then, it has been widely investigated as a hemostatic agent in diverse clinical settings, including trauma and surgical bleeding [11,12] as well as postpartum hemorrhage [13,14]. Beyond its hemostatic indications, rhFVIIa has also been used off-label as a reversal agent for anticoagulation therapy, including cases of warfarin intoxication [15] and bleeding associated with low-molecular-weight heparin [16]. Experimental and clinical studies have shown that rhFVIIa can enhance thrombin generation on activated platelets [17,18], shorten coagulation times [19], and reduce transfusion requirements [20,21]. However, its efficacy in trauma is inconsistent, and concerns about thromboembolic complications have limited its widespread adoption [22,23].

In contrast, tranexamic acid (TXA), an antifibrinolytic agent, has demonstrated a clear survival benefit for trauma patients [24]. The Clinical Randomisation of an Antifibrinolytic in Significant Hemorrhage 2 (CRASH-2) trial established TXA as a low-cost, widely available intervention that reduces trauma-related mortality without increasing thrombotic risk when administered within 3 hours of injury [25,26]. Furthermore, the beneficial hemostatic effects of TXA were demonstrated in a murine trauma model, in which periprocedural administration significantly decreased bleeding, with a marked correction of TIC and suppression of fibrinolysis [27]. These findings demonstrated that TXA not only limits hemorrhage but also restores coagulation homeostasis, providing experimental evidence that supports and extends clinical observations.

Since murine models of liver laceration provide a cost-effective and clinically relevant platform for investigating the pathophysiology of trauma-induced hemorrhage and evaluating hemostatic therapies [27,28], we directly compared the effects of rhFVIIa (a procoagulant agent) with those of TXA, which has been shown to correct TIC and has antifibrinolytic properties.

Here, we employed our established murine liver laceration model [27,28] to compare the effects of rhFVIIa with those of TXA on hemorrhage control, coagulation parameters, fibrinolysis, systemic inflammation, survival, and thrombotic complications.

## 2 Methods

### 2.1 Animal models

C57BL/6J mice were bred and maintained at the University of California San Diego internal breeding facility. Both male and female mice, aged 8 to 10 weeks, were used in the experiments. All animal protocols were approved by the Institutional Animal Care and Use Committee of the University of California San Diego.

### 2.2 Murine liver laceration model for traumatic hemorrhage

Traumatic bleeding was induced in mice by liver laceration as previously described [[27], [28], [29]]. In brief, mice approximately 8 to 10 weeks of age were anesthetized with 1.5% isoflurane and 2 L/min of oxygen, and positioned supine on a metal board for stabilization. Mice received a subcutaneous administration of 3.25 mg/kg of Ethiqa (Fidelis Animal Health) before undergoing surgery. The abdominal cavity was accessed through a midline laparotomy. Three preweighed pieces of filter paper, each approximately 0.035 g, were placed into the abdominal cavity, followed by the excision of 75% of the left liver lobe using sharp scissors (Fine Science Tools). The abdominal skin was meticulously closed with wound clips (AutoClip Kit; Fine Science Tools) to prevent any blood leakage. After 60 minutes, the wound clips were removed, and the blood-soaked filter papers were collected in preweighed tubes filled with saline to prevent them from drying out. An additional preweighed filter paper was used to absorb any residual blood present in the abdominal cavity. Blood loss was assessed by weighing the blood-soaked filter papers, with results expressed as microliters per gram of mouse body weight. A schematic representation is shown in Supplementary Figure S1

### 2.3 Administration of rhFVIIa and TXA

rhFVIIa (NovoSeven; Novo Nordisk A/S), in lyophilized powder form, was dissolved in a specified volume of histidine diluent provided by the manufacturer. After reconstitution, rhFVIIa at a dose of 3 mg/kg (because the association of human rhFVIIa with murine tissue factor [TF] is approximately 3 orders of magnitude weaker than its binding to human TF, a higher dose of 3 mg/kg was selected to achieve pharmacologically relevant activity in mice [30]) was administered as a single bolus intravenous (retro-orbital) injection 5 minutes before liver laceration. TXA (Auromedics) was prepared at a dose of 10 mg/kg (murine dosing was selected to simulate clinical dosing in trauma [1 g intravenously; ∼10-20 mg/kg]). Accordingly, a dose of 10 mg/kg TXA was used, consistent with prior murine studies of traumatic brain injury [31,32] and liver laceration [27], where it was administered safely and conferred a survival benefit, diluted in 0.9% injectable-grade saline, and administered as a single bolus intravenous (retro-orbital) injection 5 minutes prior to the liver laceration procedure. The control mice received an equal volume of 0.9% injectable-grade saline (∼100 μL).

### 2.4 Survival study

In the survival model, blood loss was assessed 60 minutes post liver laceration, followed by closure of the abdominal incision with sterile sutures or wound clips (AutoClip Kit; Fine Science Tools) and sealed with LiquiVet Rapid Tissue Adhesive (Oasis Medical). Mice were administered 400 μL of saline subcutaneously before being returned to their cages, with an additional daily injection of 400 μL of saline for the first 3 days. Blood samples were collected (∼100 μL) through retro-orbital access at 6 hours after liver laceration and processed as described in Section 2.5. After 72 hours, a second dose of 3.25 mg/kg of Ethiqa was administered subcutaneously to manage pain. Mice were subsequently monitored during recovery in accordance with Institutional Animal Care and Use Committee-approved postoperative care guidelines.

### 2.5 Collection and processing of blood samples

Blood was collected via retro-orbital sampling into a 3.8% sodium citrate solution (blood-to-anticoagulant ratio, 9:1) 60 minutes or 6 hours after trauma (different groups of mice). The samples were centrifuged at 2000 × *g* for 10 minutes, followed by a second centrifugation at 13,500 × *g* for 5 minutes to obtain platelet-poor plasma. The resulting plasma was used for coagulation assays and other downstream analysis. All plasma samples were stored at −80 °C until further use.

### 2.6 Assessment of the activity levels of coagulation factors

All coagulation times were measured using an ST4 coagulometer (Diagnostica Stago). To ensure the absence of interference of rhFVIIa and TXA in the assays, mouse plasma collected at baseline and after liver laceration (saline-treated) was spiked *ex vivo* with rhFVIIa or TXA across a range of concentrations and analyzed using activated partial thromboplastin time (aPTT) and FV activity assays at varying plasma dilutions (Supplementary Methods). *Ex vivo* spiking demonstrated that rhFVIIa induced dilution-dependent interference in FV activity assays at low plasma dilutions, which was mitigated at higher dilutions, whereas TXA did not affect aPTT or FV activity measurements under the conditions tested (Supplementary Figure S2A–F). aPTT was determined by mixing 25 μL mouse plasma with 25 μL aPTT Reagent (Diagnostica Stago), followed by the addition of 25 μL CaCl_2_ (25 mM) in HEPES-buffered saline (20 mM HEPES, 147 mM NaCl, 3 mM KCl, pH 7.4) after 3 minutes at 37 °C, as described previously [33]. To determine the activity levels of various coagulation factors, plasma samples from TXA-treated mice were diluted 1:100 with dilution buffer, and samples from rhFVIIa-treated mice were diluted 1:150 to mitigate assay interference. Diluted murine plasma (5 μL) was mixed with FII-, FV-, or FX-deficient plasma (20 μL; Enzyme Research Laboratories) and HEPES-buffered saline containing 0.5% bovine serum albumin (25 μL; Sigma-Aldrich), and incubated at 37 °C for 1 minute. The clotting time was recorded following the addition of Innovin (25 μL; DADE Behring). FVIII chromogenic assays were performed using the chromogenix coamatic factor VIII assay kit (DiaPharma) according to the manufacturer’s protocol. Fibrinogen concentration was measured using the Clauss method [34]. Assays were performed in separate runs, and interassay variability was controlled by including previously analyzed samples in each run to ensure reproducibility and reliable comparisons across groups.

### 2.7 Plasma biomarker analysis

Plasma biomarkers, including the thrombin-antithrombin (TAT) complex (Siemens Healthcare), tissue-type plasminogen activator (tPA; Innovative Research), plasmin-α2-antiplasmin (PAP) complex (Novus Biologicals), and D-dimer (specifically recognizes the degradation product D-dimer of cross-linked fibrin; catalog number EEL094, Invitrogen), were measured using commercially available kits according to the manufacturer’s instructions.

### 2.8 Tissue harvest and staining

Lung tissues were harvested at baseline and after trauma from mice treated with saline, rhFVIIa, or TXA on days 1, 2, and 7 post liver laceration, after being euthanized using CO_2_ [35]. The tissues were fixed in Z-Fix (Anatech) for 20 to 24 hours, processed, and paraffin-embedded. The paraffin-embedded tissues were sectioned (4 μm thickness) and stained with hematoxylin-eosin and with orcein and martius scarlet blue for fibrin, as described previously [36]. The images were captured on a NanoZoomer 2.0-HT brightfield slide scanner at 20× magnification (Hamamatsu Photonics).

### 2.9 Plasma cytokine analysis

Plasma cytokine concentrations were measured using a commercially available kit at baseline and at 60 minutes and 6 hours after liver laceration (15-Plex Q-Plex Mouse Cytokine Inflammation High Sensitivity Kit; Quansys Biosciences; interferon γ, interleukin (IL)-1α, IL-1β, IL-2, IL-3, IL-4, IL-5, IL-6, IL-10, IL-12p70, IL-17A, macrophage inflammatory protein-1α, granulocyte macrophage-colony stimulating factor, tumor necrosis factor-α, and regulated on activation normal T-cell expressed and secreted). The assay was performed according to the manufacturer's instructions, and data were analyzed using the manufacturer’s Q-View software.

### 2.10 Statistical analysis

The sample sizes exhibited a nonnormal distribution. Consequently, the data were represented as medians with IQRs. Group comparisons were performed using the nonparametric Mann–Whitney U-test. Survival analyses were conducted using Kaplan–Meier survival curves with differences evaluated by the Mantel–Cox log-rank test. Statistical analyses were performed with GraphPad Prism version 7.0 (GraphPad Software).

## 3 Results

### 3.1 Management of hemorrhage after liver laceration with rhFVIIa and TXA

**Figure 1:**
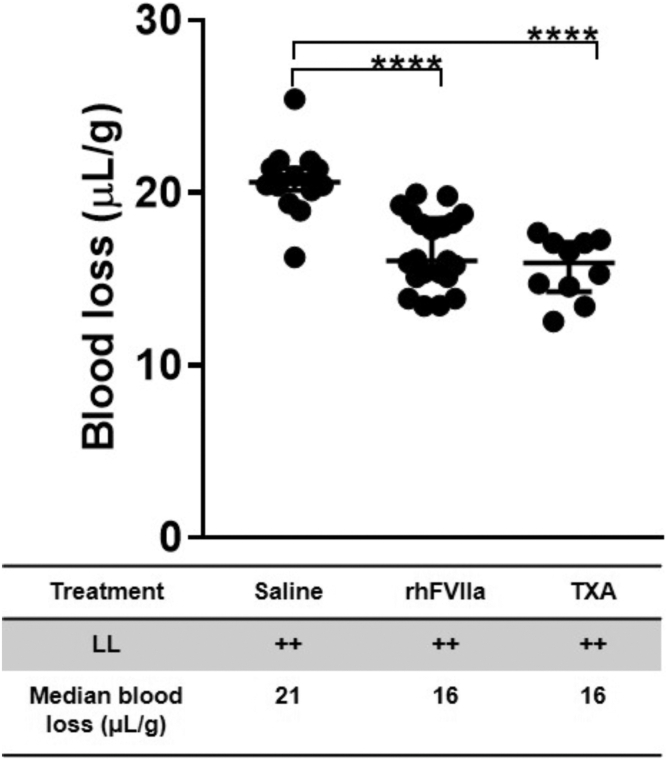
Blood loss associated with liver laceration (LL). Severe hemorrhage was induced in C57BL/6J mice by midline laparotomy followed by LL. Mice received periprocedural treatment with saline (100 μL), recombinant human activated factor VII (rhFVIIa; 3 mg/kg), or tranexamic acid (TXA; 10 mg/kg); *n* = 7 to 21 per group. Blood loss was quantified 60 minutes posttrauma. Statistical comparisons were performed using the nonparametric Mann–Whitney U-test. Data are shown as medians with IQRs, with horizontal bars indicating group medians; ∗∗∗∗*P* ≤ .0001.

Severe hemorrhage induced by laparotomy and liver laceration resulted in substantial blood loss at 60 minutes (21 μL/g; *n* = 15). Periprocedural administration of rhFVIIa (3 mg/kg) or TXA (10 mg/kg) significantly reduced blood loss to comparable levels (16 μL/g; *P* ≤ .0001; *n* = 10-21 per group; Figure 1). These findings indicate that periprocedural rhFVIIa and TXA are equally effective in reducing blood loss, with neither treatment reducing bleeding below a threshold of ∼15 μL/g. This threshold of bleed reduction has been observed previously with TXA [27].

### 3.2 Coagulopathy after liver laceration and the effects of rhFVIIa and TXA

Since rhFVIIa and TXA resulted in similar reductions in bleeding after liver laceration, their effects on coagulopathy were evaluated. The presence of general coagulopathy was assessed by aPTT, thrombin generation (TAT complexes), and fibrinogen levels. TIC [37] was assessed specifically for decreases in FV and FVIII activity levels to determine whether TIC or disseminated intravascular coagulation (DIC) was predominant. In DIC, global clotting factor consumption would predominate, delineated by additional decreases in FII and FX [38].

**Figure 2:**
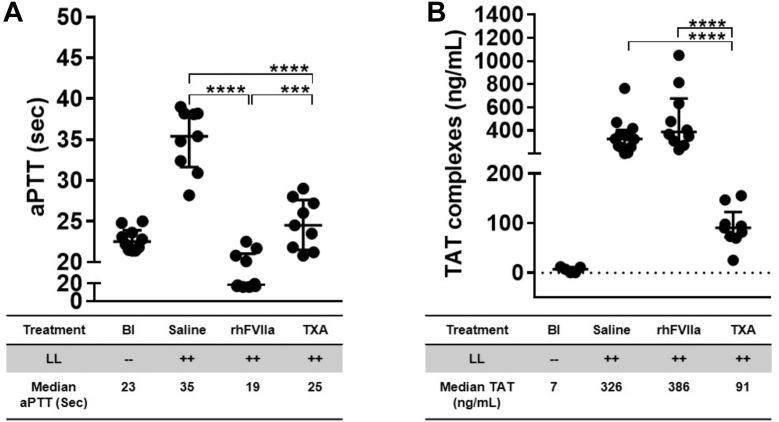
General coagulopathy following liver laceration (LL). Severe hemorrhage was induced in mice by midline laparotomy followed by LL. Mice received periprocedural treatment with saline (100 μL), recombinant human activated factor VII (rhFVIIa; 3 mg/kg), or tranexamic acid (TXA; 10 mg/kg); *n* = 7 to 21 per group. Coagulation parameters were evaluated 60 minutes posttrauma. The following outcomes were assessed: (A) activated partial thromboplastin time (aPTT) and (B) thrombin-antithrombin (TAT) complex levels. Statistical comparisons were performed using the nonparametric Mann–Whitney U-test. Data are presented as medians with IQRs, with horizontal bars indicating group medians; ∗∗∗*P* ≤ .001, ∗∗∗∗*P* ≤ .0001. Bl, baseline; Sec, seconds.

Hemorrhage induced by liver laceration resulted in the development of coagulopathy. There was a significant prolongation of aPTT from 23 seconds at baseline (*n* = 10) to 35 seconds 60 minutes after liver laceration (*P* ≤ .0001; *n* = 10). rhFVIIa shortened the aPTT below baseline (19 seconds; *P* ≤ .0006; *n* = 10), while TXA normalized the aPTT (25 seconds; *n* = 10; Figure 2A). The reduction in aPTT noted with rhFVIIa was significantly more pronounced than that with TXA (*P* ≤ .0005). TAT complexes were significantly increased after liver laceration (326 ng/mL; *P* ≤ .0003; *n* = 12), which was further exacerbated by rhFVIIa (386 ng/mL; *P* ≤ .0001; *n* = 10). In contrast, TXA markedly decreased TAT complex formation (91 ng/mL; *P* ≤ .0001; *n* = 9; Figure 2B). These findings suggest that despite similar reductions in bleeding, rhFVIIa and TXA demonstrated divergent effects on aPTT and thrombin generation.

**Figure 3:**
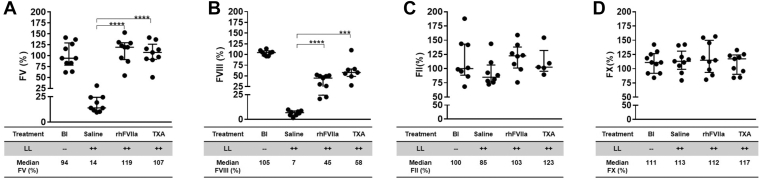
Assessment of trauma-induced coagulopathy (TIC) following liver laceration (LL). Development of TIC was assessed in mice subjected to severe bleeding induced by midline laparotomy and LL. Mice received periprocedural treatment with saline (100 μL), recombinant human activated factor (F)VII (rhFVIIa; 3 mg/kg), or tranexamic acid (TXA; 10 mg/kg); *n* = 8 to 15 per group. Coagulation factor activity levels were measured 60 minutes posttrauma. (A) FV, (B) FVIII, (C) FII, and (D) FX. Statistical comparisons were performed using the nonparametric Mann–Whitney U-test. Data are presented as medians with IQRs, with horizontal bars indicating group medians; ∗∗∗*P* ≤ .001, ∗∗∗∗*P* ≤ .0001. Bl, baseline.

To evaluate the effects of rhFVIIa and TXA on TIC and DIC, we measured the activity levels of FV, FVIII, FII, FX, and fibrinogen following liver laceration. Liver laceration resulted in a significant reduction in FV and FVIII activity levels, while FII and FX levels remained normal, underscoring that TIC, not DIC, was the predominant form of coagulopathy, as previously observed [27,28] (Figure 3A–D). Both rhFVIIa and TXA effectively prevented a reduction in FV activity (rhFVIIa and TXA∼100%; *n* = 7-9; Figure 3A) and partially restored FVIII activity (rhFVIIa and TXA ∼50%; *n* = 7-9; Figure 3B).

### 3.3 Fibrinolysis after liver laceration and the effects of rhFVIIa and TXA

Traumatic bleeding is often accompanied by hyperfibrinolysis, leading to exacerbated blood loss and impaired hemostasis [39]. The extent of fibrinolysis after liver laceration was assessed by fibrinogen, tPA, PAP complexes, and D-dimer.

**Figure 4:**
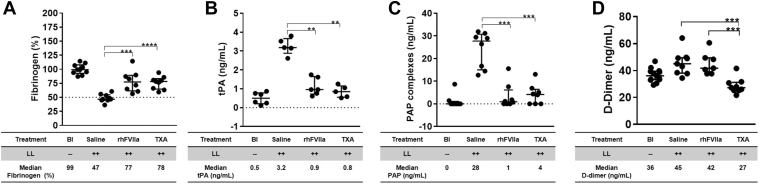
Fibrinolysis after liver laceration (LL). Severe hemorrhage was induced by midline laparotomy followed by LL involving the removal of ∼75% of the left liver lobe. Mice received periprocedural treatment with saline (100 μL), recombinant human activated factor VII (rhFVIIa; 3 mg/kg), or tranexamic acid (TXA; 10 mg/kg); *n* = 8 to 14 per group. Plasma samples were collected 60 minutes posttrauma to evaluate fibrinolytic activity. Shown are levels of (A) fibrinogen, (B) tissue-type plasminogen activator (tPA), (C) plasmin-α2-antiplasmin (PAP) complexes, and (D) D-dimer. Statistical comparisons were performed using the nonparametric Mann–Whitney U-test. Data are presented as medians with IQRs, with horizontal bars indicating group medians; ∗∗*P* ≤ .01, ∗∗∗*P* ≤ .001, ∗∗∗∗*P* ≤ .0001. Bl, baseline.

Liver laceration resulted in ∼50% depletion of fibrinogen (*P* ≤ .0001; *n* = 9). Both rhFVIIa and TXA resulted in partial correction, reducing fibrinogen depletion to ∼25% (*P* ≤ .001; *n* = 8-9 per group; Figure 4A). There was notable activation of the fibrinolytic system, evidenced by marked increases in tPA, PAP complexes, and D-dimer formation. tPA levels rose to 3.9 ng/mL (*P* ≤ .001; *n* = 5) and were significantly reduced to 0.9 or 0.8 ng/mL (*P* ≤ .001; *n* = 5-6 per group) by rhFVIIa or TXA, respectively (Figure 4B). Similarly, PAP complex formation was markedly elevated (28 ng/mL; *P* ≤ .0002; *n* = 8) and effectively suppressed by both rhFVIIa (1 ng/mL; *P* ≤ .001; *n* = 8) and TXA (4 ng/mL; *P* ≤ .001; *n* = 8; Figure 4C). D-dimer formation was also elevated (45 ng/mL; *P* ≤ .01; *n* = 9) but was significantly suppressed only by TXA (27 ng/mL; *P* ≤ .001; *n* = 9), not by rhFVIIa (42 ng/mL; *n* = 7; Figure 4D).

These findings demonstrate the biological effects of TXA on fibrinolysis and support a mechanistic basis for its antifibrinolytic action rather than assay interference, consistent with *in vitro* spiking experiments [40] and prior *in vivo* studies [41].

### 3.4 Effects of rhFVIIa and TXA on survival and pulmonary thrombotic complications following liver laceration

**Figure 5:**
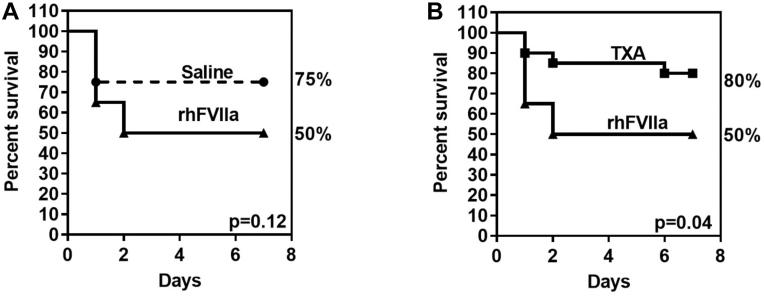
Survival study after liver laceration. Severe hemorrhage was induced by midline laparotomy followed by liver laceration involving the removal of ∼75% of the left liver lobe. Mice received periprocedural treatment with saline (100 μL), recombinant human activated factor VII (rhFVIIa; 3 mg/kg), or tranexamic acid (TXA; 10 mg/kg); *n* = 20 per group. Blood loss was quantified 60 minutes posttrauma by weighing blood-soaked sponges placed in the abdominal cavity. The abdominal incision was closed with sutures/wound clips and tissue adhesive, and the mice were returned to their cages. Supportive care consisted of daily administration of saline (400 μL) for the first 3 days. Survival was monitored for 7 days posttrauma. (A) Saline- and rFVIIa-treated groups and (B) TXA- and rhFVIIa-treated groups.

Most mice treated with either saline or TXA survived the traumatic injury (7-day survival: 75% or 80%, respectively; *n* = 20 per group), while survival with rhFVIIa was only 50% (*n* = 20). The survival difference between TXA- and rhFVIIa-treated mice was significant (*P* = .04; Figure 5A, B).

**Figure 6:**
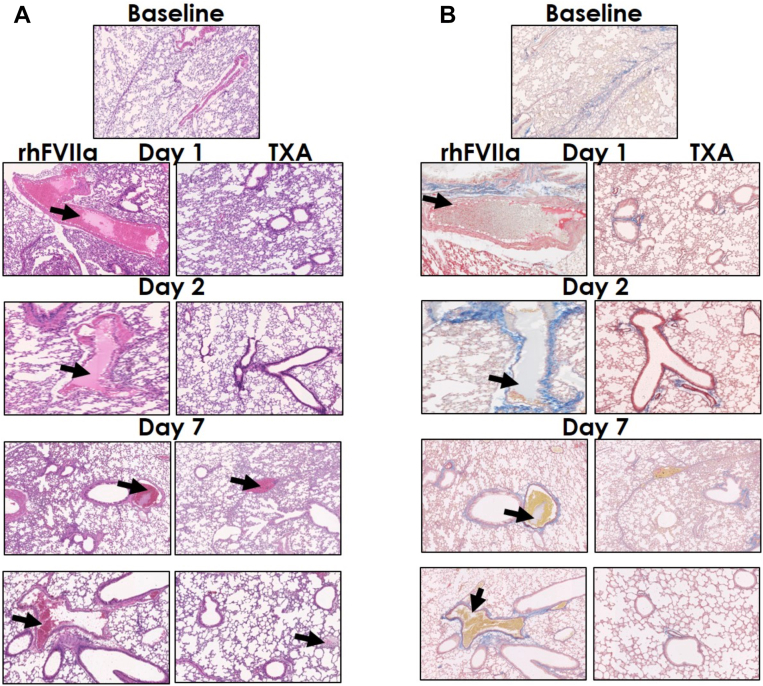
Pulmonary histological analysis following liver laceration. Severe hemorrhage was induced by midline laparotomy followed by liver laceration involving the removal of ∼75% of the left liver lobe. Mice received periprocedural treatment with saline (100 μL), recombinant human activated factor VII (rhFVIIa; 3 mg/kg), or tranexamic acid (TXA; 10 mg/kg); *n* = 4 to 10 per group. Lung tissues were collected at baseline and on days 1, 2, and 7 posttrauma. Paraffin-embedded sections (4 μm) were deparaffinized and subjected to hematoxylin-eosin and orcein and martius scarlet blue staining. Slides were imaged using a Hamamatsu NanoZoomer brightfield slide scanner at 20× magnification. (A) Representative hematoxylin-eosin–stained lung sections from baseline and on days 1, 2, and 7 across treatment groups. (B) Representative orcein and martius scarlet blue-stained lung sections from baseline and on days 1, 2, and 7 across treatment groups. Black arrows indicate preformed fibrin or microthrombi.

To evaluate pulmonary thrombotic contributions to mortality, surviving mice treated periprocedurally with saline, rhFVIIa, or TXA were euthanized on days 1, 2, and 7 to examine lung tissue. Hematoxylin-eosin staining revealed the presence of pulmonary microthrombi and preformed fibrin (preformed fibrin refers to fibrin that has already assembled into its polymeric structure prior to being cross-linked and stabilized) [42] in rhFVIIa-treated mice on day 1 (*n* = 5/5), day 2 (*n* = 4/4), and day 7 (*n* = 5/10; Figure 6A). These findings were further corroborated by staining with orcein and martius scarlet blue. In contrast, TXA-treated mice exhibited no evidence of pulmonary microthrombi or preformed fibrin on day 1 (*n* = 0/4) and day 2 (*n* = 0/4), with minimal preformed fibrin observed in 2 mice on day 7 (*n* = 2/6; Figure 6B). There was no microthrombi or preformed fibrin present in saline-treated mice (day 1, *n* = 0/5; day 2, *n* = 0/4; and day 7, *n* = 0/5).

### 3.5 Effects of rhFVIIa and TXA on cytokine profiles after liver laceration

**Figure 7:**
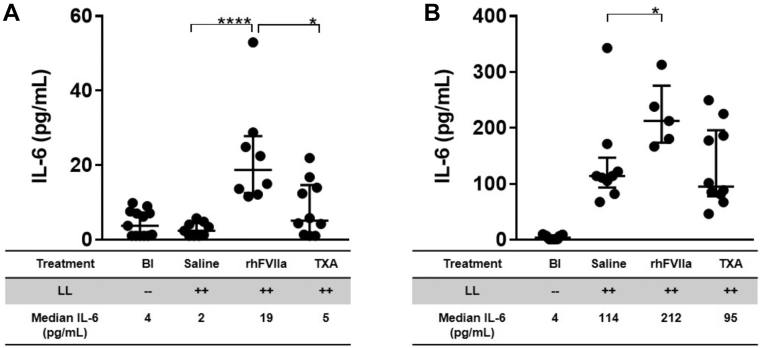
Plasma interleukin (IL)-6 levels following liver laceration (LL). Plasma IL-6 concentrations were measured at baseline and at 60 minutes and 6 hours after induction of severe bleeding by midline laparotomy and LL. Mice received prophylactic treatment with saline (100 μL), recombinant human activated factor VII (rhFVIIa; 3 mg/kg), or tranexamic acid (TXA; 10 mg/kg); *n* = 5 to 13 per group. IL-6 levels are shown at (A) 60 minutes and (B) 6 hours posttrauma. Statistical comparisons were performed using the nonparametric Mann–Whitney U-test. Data are presented as medians with IQRs, with horizontal bars indicating group medians; ∗*P* ≤ .05, ∗∗∗∗*P* ≤ .0001. Bl, baseline.

To investigate whether rhFVIIa or TXA influences inflammatory effects after trauma, we quantified 14 cytokines (IL-1α, IL-1β, IL-2, IL-3, IL-4, IL-6, IL-10, IL-12p70, IL-17, monocyte chemoattractant protein-1, macrophage inflammatory protein-1α, granulocyte macrophage-colony stimulating factor, and tumor necrosis factor-α, and regulated on activation normal T-cell expressed and secreted) in plasma at baseline and at 1 and 6 hours after liver laceration, the last time point before the onset of mortality. Of the 14 cytokines measured, only IL-6 was modulated by treatment. An early rise in IL-6 was detected exclusively in rhFVIIa-treated mice at 1 hour post liver laceration (19 pg/mL; *P* ≤ .004; *n* = 9; Figure 7A). By 6 hours post liver laceration, IL-6 concentrations were markedly elevated in all groups relative to baseline, but were approximately twice as high with rhFVIIa (212 pg/mL; *n* = 5) compared with saline or TXA (114 or 95 pg/mL, respectively; *n* = 9-10 mice per group; *P* ≤ .0002; Figure 7B). These findings suggest that rhFVIIa induces an early and pronounced inflammatory response to trauma.

## 4 Discussion

This murine trauma study demonstrates that rhFVIIa effectively reduces blood loss after traumatic injury but at the expense of excessive procoagulant activity, thrombo-inflammatory responses, pulmonary thromboembolic complications, and increased mortality. These findings align with previous clinical observations of human trauma patients, where rhFVIIa curbed bleeding and reduced transfusion requirements but increased thromboembolic events without improving survival [22,23]. Ultimately, these detrimental effects limited the usefulness of rhFVIIa in trauma management. These studies were conducted 2 decades ago and dampened enthusiasm for developing procoagulant molecules for hemostatic interventions after trauma, leaving a gap and an unmet medical need for targeted intervention.

In contrast to rhFVIIa, the antifibrinolytic agent TXA has been shown to reduce mortality in trauma patients when administered early [25,43]. Consistent with clinical evidence, TXA reduced blood loss in the murine trauma setting presented here, as previously shown [27], and, unlike rhFVIIa, corrected TIC, as demonstrated by normalization of aPTT and partial restoration of FV and FVIII activity, without accelerating inflammation (lower IL-6 levels at 6 hours), thromboembolic complications (no pulmonary microthrombi or preformed fibrin during the early posttraumatic period), or compromising survival.

Moreover, in contrast to TXA, rhFVIIa did not suppress D-dimer formation after liver laceration, thereby elevating levels of tPA, PAP, and D-dimer relative to baseline, consistent with enhanced fibrinolytic activity following injury. However, both rhFVIIa and TXA suppressed tPA and PAP levels. We speculate that this reduction reflects mitigation of trauma-induced endothelial activation and tPA release, with a consequent decrease in plasminogen activation and PAP formation [44]. Taken together, these observations suggest that rhFVIIa was unable to suppress D-dimer formation despite apparent inhibition of systemic plasmin generation, suggesting persistent fibrin turnover that may involve thrombo-inflammatory or localized protease-mediated pathways [39]. Neutrophil-derived serine proteases, particularly neutrophil elastase and cathepsin G, cleave cross-linked fibrin and generate D or D-like fragments that are detected by D-dimer immunoassays [[45], [46], [47]]. To this end, rhFVIIa exacerbated IL-6 elevation during the first 6 hours after trauma (clinically defined as the early stage of TIC [39]), accelerating the development of a pronounced inflammatory response, which can be associated with protease-driven fibrin degradation [48,49]. The rhFVIIa-induced thrombo-inflammatory environment during early TIC may aggravate hypercoagulability during late TIC (clinically defined as 6-24 hours posttrauma), leading to more thromboembolic complications than expected in humans and animal models alike [22,23,50]. Of note, although the plasma half-life of rhFVIIa is short (approximately 2 hours in humans [22,51]), its binding to endothelial protein C receptor enables transendothelial movement and accumulation in extravascular tissues, where functionally active rhFVIIa may persist far longer than in circulation [52]. This is problematic, given the already high incidence of venous thromboembolism in the posttraumatic setting, where early (<48 hours) initiation of venous thromboembolism chemoprophylaxis, despite the associated bleeding risk, is often considered necessary to mitigate thromboembolic complications after solid organ injury [53,54].

The conversion of hypocoagulable early TIC to hypercoagulable late TIC is mechanistically poorly understood [5,39,55]; however, our observations suggest that sustained thrombin generation may be a contributing mechanism, potentially through downstream thrombo-inflammatory effects, such as neutrophil activation and NETosis (neutrophil extracellular trap formation) [49,56,57]. Effects may be amplified by rhFVIIa, as evidenced by accelerated TAT complex formation (Figure 2B). It is also possible that rhFVIIa-driven thrombin generation initiates neutrophil protease activity locally within pulmonary microthrombi [22,48,58], contributing to residual D-dimer despite suppression of systemic plasmin activity. Thus, there is good reason to suspect that elevated D-dimer in rhFVIIa-treated mice may reflect thromboinflammatory fibrin turnover rather than classical plasmin-mediated fibrinolysis. In contrast, TXA significantly reduced D-dimer, consistent with direct inhibition of plasmin-mediated fibrin degradation [59,60], as demonstrated in our previous studies examining the effects of TXA on TIC correction in anemic and nonanemic mice [27]. Histological analyses of murine lungs during the posttraumatic period further substantiate the interpretation of the hemostatic findings. Pulmonary microthrombi and preformed fibrin developed only in rhFVIIa-treated mice, most prominently between days 1 and 2, coinciding with the late phase of TIC [9], and were associated with early mortality, consistent with prior observations of human trauma [2,19]. These late-stage effects of rhFVIIa are noteworthy, as they imply that rhFVIIa may persist within or interact with the subendothelial compartment for an extended period of time. In contrast, TXA- and saline-treated mice showed minimal pulmonary thrombotic changes. Together, these observations support a mechanistic link between excessive thrombin generation, systemic fibrin deposition, and organ injury as drivers of rhFVIIa-associated mortality.

Trauma-associated mortality because of uncontrolled posttraumatic bleeding remains the major cause of death below age 45 [61,62]. TIC aggravates bleeding, develops in approximately one-third of trauma patients and approximately two-thirds of patients with traumatic brain injury, and almost always results in death [39,55]. However, there has been little effort to develop targeted therapies to intercept TIC beyond optimizing transfusion support and fluid resuscitation [55,63]. There is a critical need for new targeted interventions that correct TIC without exacerbating thromboembolic or inflammatory complications. Emerging preclinical strategies, including CT-001 [50,64] and ^Super^FVa, are designed to target pathogenic mechanisms of TIC, such as excessive activated protein C-mediated inactivation of FV and FVIII, while limiting systemic procoagulant exposure [7,28,39,63,65]. However, while emphasizing these strategies, it should be kept in mind that TIC is a multifactorial process, as described by Cap et al. [66].

This study has several limitations. First, periprocedural administration of hemostatic agents does not entirely reflect clinical practice, in which these therapies are given after bleeding onset. However, given the close proximity of the traumatic injury (a few minutes prior), we believe that extrapolating to early treatment effects is reasonable. Second, the dose of 3 mg/kg rhFVIIa, selected to achieve hemostasis in murine models, is higher than those typically used in clinical practice (90 μg/kg). Higher doses of rhFVIIa are necessary in murine models to achieve comparable hemostatic efficacy, as human rFVIIa interacts with murine TF with reduced binding affinity and catalytic efficiency compared with its interaction with human TF [30,[67], [68], [69]]. Therefore, 3 mg/kg is the lowest dose of rhFVIIa widely used to achieve hemostasis in injury models.

## 5 Conclusions

In conclusion, rhFVIIa and TXA both reduced blood loss after liver laceration, but only TXA provided a survival benefit without thromboembolic complications. rhFVIIa was associated with excessive thrombin generation, early IL-6 induction, pulmonary microthrombi, and early mortality, highlighting its potential risks in trauma. These findings highlight the need for next-generation, targeted hemostatic agents to restore hemostasis while minimizing widespread activation of the coagulation and inflammatory systems beyond the site of injury.
