# Supplementary material for: The effects of recombinant human activated factor VII and tranexamic acid on traumatic bleeding and mortality in mice
Source: Res Pract Thromb Haemost. 2026 Mar 26;10(3):103436. doi: 10.1016/j.rpth.2026.103436 (PMC13100279; doi:10.1016/j.rpth.2026.103436)
Supplement: Supplementary Figures [file mmc1.docx]

**Supplementary Information**

**The effects of recombinant human activated FVII and tranexamic acid on traumatic bleeding and mortality in mice**

Bilgimol Chumappumkal Joseph, Juan Andres De Pablo-Moreno, Nicca Falah, Abraham Wentzel, Mia Lora Cacho, Eduardo Frias-Anaya, Miguel A. Lopez-Ramirez, Annette von Drygalski

**
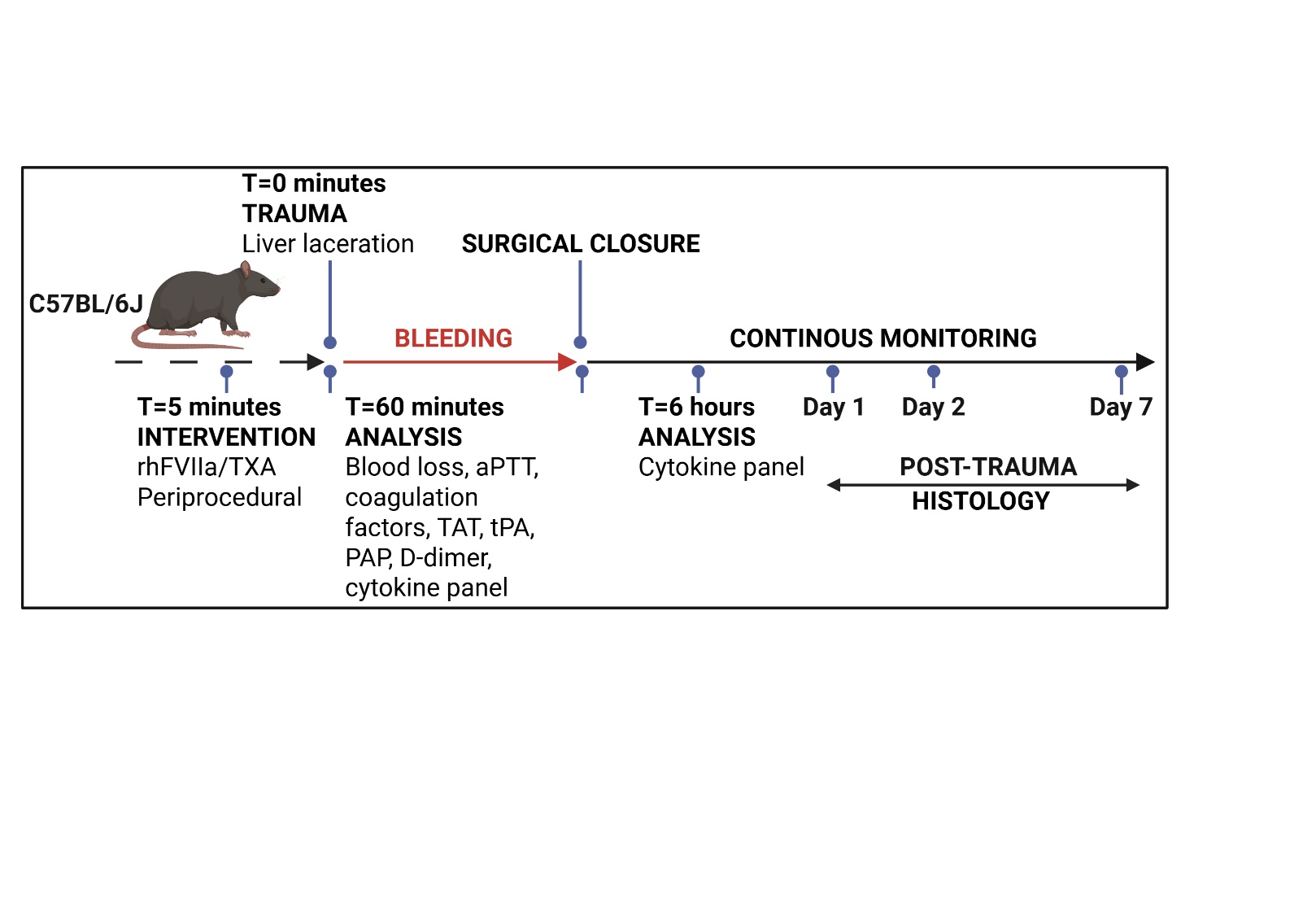
**

**Supplementary Figure S1. Overview of the liver laceration model and experimental timeline.** Mice underwent midline laparotomy followed by liver laceration (~75% of the left liver lobe). Mice received either saline (100 µL, vehicle control), rhFVIIa (3 mg/kg), or TXA (10 mg/kg administered intravenously (retro-orbital) 5 minutes prior to liver laceration. Pre-weighed sponges were placed in the abdominal cavity to absorb blood, retrieved 60 minutes post-trauma, and weighed to quantify blood loss. The abdomen was closed with sutures or wound clips and tissue adhesive. Blood samples were collected at 60 minutes and 6 hours post-trauma by retro-orbital access. Plasma was analyzed for coagulation parameters (aPTT, coagulation factor activity, TAT complexes, tPA, PAP complexes, and D-dimer) at 60 minutes, and for cytokines at both 60 minutes and 6 hours. Survival was monitored for 7 days, and lung tissue was collected on days 1, 2, and 7 for histological analysis.

aPTT, Activated partial thromboplastin time; D-Dimer, D fragment of fibrin; PAP, plasmin-alpha-2-antiplasmin; rhFVIIa, recombinant human activated factor VII; TAT, Thrombin anti-thrombin; TXA, Tranexamic Acid; T, Time; tPA, tissue plasminogen activator.


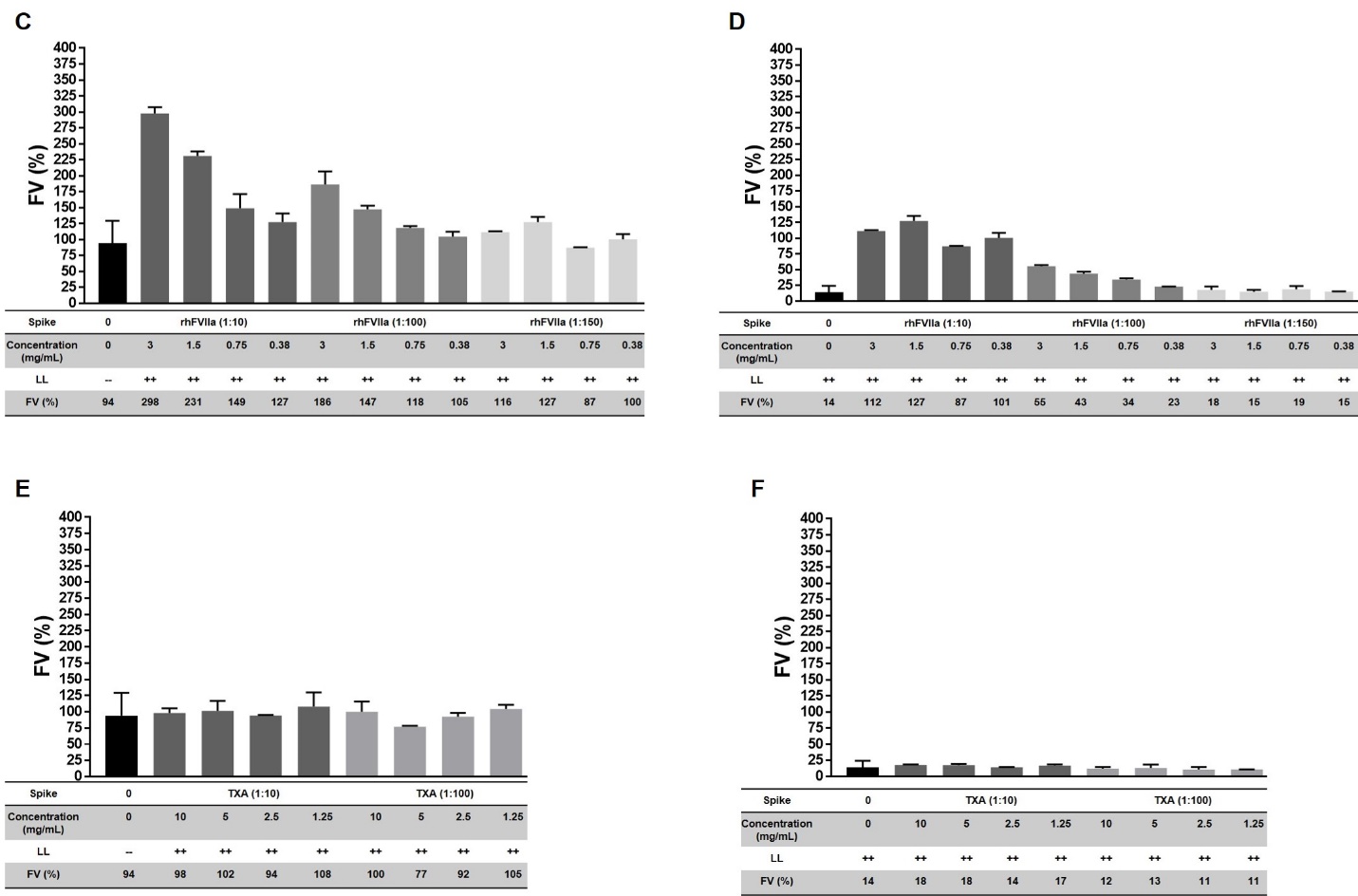

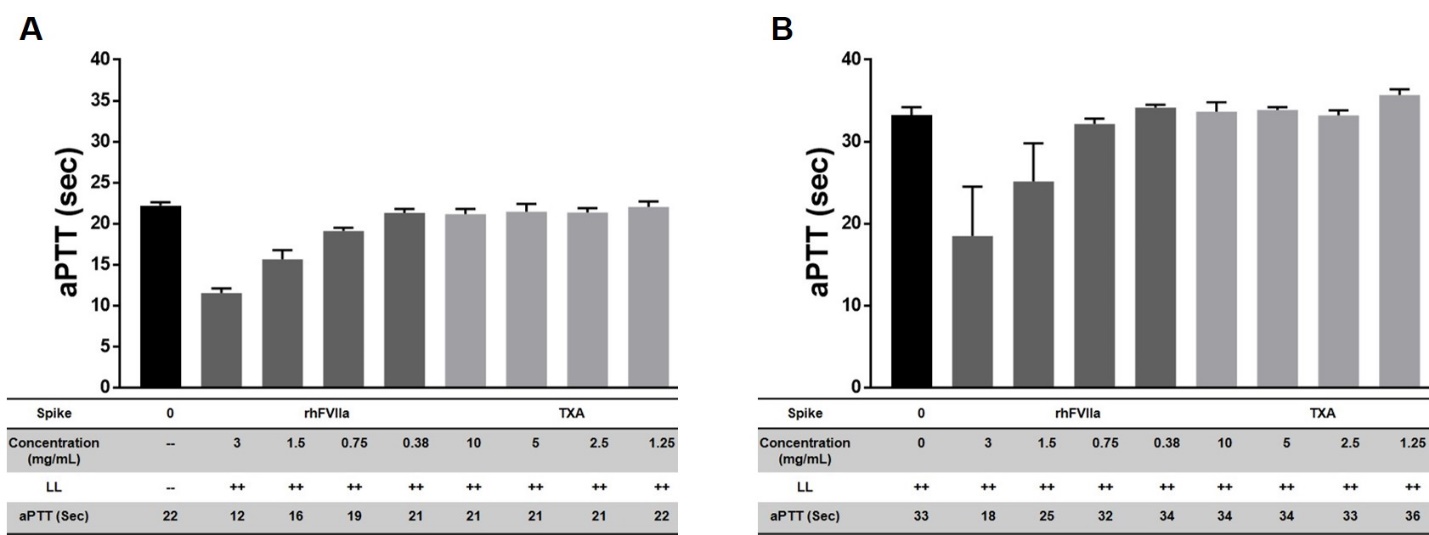


**Supplementary Figure S2. Effects of rhFVIIa and TXA on aPTT and FV activity assays in mouse plasma.** (A) aPTT measured in baseline citrated mouse plasma before and after ex vivo spiking with increasing concentrations of rhFVIIa) (3, 1.5, 0.75, and 0.38 mg/mL) or TXA (10, 5, 2.5, and 1.25 mg/mL). (B) aPTT measured in plasma collected after liver laceration without treatment (saline-treated) before and after spiking with rhFVIIa or TXA at the same concentrations. (C) FV activity measured in baseline plasma spiked with rhFVIIa at indicated concentrations and plasma dilutions (1:10, 1:100, 1:150). (D) FV activity measured in liver laceration saline-treated plasma spiked with rhFVIIa at the same concentrations and dilutions as in (C). (E) FV activity measured in baseline plasma spiked with TXA at indicated concentrations and plasma dilutions (1:10, 1:100). (F) FV activity measured in liver laceration saline-treated plasma spiked with TXA at the same concentrations and dilutions as in (E). aPTT was measured using a standard clot-based assay without plasma dilution. FV activity was determined using a one-stage clot-based assay with FV-deficient plasma and tissue factor activation (Innovin).

aPTT, Activated partial thromboplastin time; FV, Factor V; LL, liver laceration; rhFVIIa, recombinant human activated factor VII; TXA, Tranexamic Acid.
