# Supplementary material for: The effects of recombinant human activated factor VII and tranexamic acid on traumatic bleeding and mortality in mice
Source: Res Pract Thromb Haemost. 2026 Mar 26;10(3):103436. doi: 10.1016/j.rpth.2026.103436 (PMC13100279; doi:10.1016/j.rpth.2026.103436)
Supplement: Supplementary Methods [file mmc2.docx]

**Supplementary Methods**

**The effects of recombinant human activated FVII and tranexamic acid on traumatic bleeding and mortality in mice**

Bilgimol Chumappumkal Joseph, Juan Andres De Pablo-Moreno, Nicca Falah, Abraham Wentzel, Mia Lora Cacho, Eduardo Frias-Anaya, Miguel A. Lopez-Ramirez, Annette von Drygalski

**Method**

**Validation of aPTT and FV activity assays in the presence of rhFVIIa and TXA**

To assess potential assay interference of rhFVIIa or TXA, ex vivo spiking and dilution experiments were performed using citrated mouse plasma collected at baseline and after liver laceration without treatment (saline-treated). Plasma samples were spiked with rhFVIIa (3, 1.5, 0.75, and 0.38 mg/mL) or TXA (10, 5, 2.5, and 1.25 mg/mL). aPTT was measured before and after spiking using a standard aPTT assay protocol without plasma dilution. Following spiking, plasma samples were diluted in assay buffer to final plasma dilutions of 1:10, 1:100, or 1:150, as specified for each experiment. FV activity was measured before and after spiking using a one-stage clot-based FV activity assay employing FV-deficient plasma and tissue factor–based activation (Innovin). Plasma dilution conditions were selected to minimize rhFVIIa- or TXA-related assay interference while preserving sensitivity to endogenous FV activity.

**Results**

**Effects of TXA and rhFVIIa on aPTT measurements**

In baseline plasma, aPTT was 22 seconds. Spiking with rhFVIIa resulted in a concentration-dependent shortening of aPTT, with clotting times decreasing to 12 seconds at the highest concentration tested. In contrast, spiking with tranexamic acid had no measurable effect on aPTT, with clotting times remaining unchanged across all concentrations (Supplementary Figure 2A).

Following liver laceration, saline-treated plasma exhibited prolonged aPTT (33 seconds). rhFVIIa spiking again produced a dose-dependent shortening of aPTT, whereas TXA spiking did not alter clotting times. These findings indicate that TXA does not interfere with aPTT measurements, while rhFVIIa predictably shortens aPTT due to its procoagulant activity (Supplementary Figure 2B).

**Dilution-dependent effects of rhFVIIa and TXA on FV activity measurements**

Baseline mouse plasma exhibited FV activity of 94%, whereas plasma collected after liver laceration without treatment showed markedly reduced FV activity (14%). Ex vivo spiking with recombinant activated factor VII resulted in a concentration- and dilution-dependent increase in apparent FV activity. At a low plasma dilution (1:10), rhFVIIa markedly increased FV activity in baseline plasma (127–231%) and normalized FV activity in plasma after liver-laceration without treatment (87–127%), consistent with assay interference under low-dilution conditions. At an intermediate dilution (1:100), rhFVIIa continued to elevate apparent FV activity in baseline plasma (105–186%) and partially increased FV activity in after liver-laceration without treatment (23–55%). In contrast, at a higher dilution (1:150), FV activity in baseline plasma returned to values near the expected physiological range (87–127%), while FV activity in after liver-laceration without treatment remained low (15–19%) across all rhFVIIa concentrations (Supplementary Figure 2C and D).

Spiking with tranexamic acid did not substantially alter FV activity measurements. In baseline plasma, FV activity remained within the expected range following TXA spiking at both 1:10 (94–108%) and 1:100 (77–105%) dilutions. In plasma after liver-laceration without treatment, FV activity remained low and comparable to unspiked controls at both 1:10 (14–18%) and 1:100 (11–13%) dilutions (Supplementary Figure 2E and F).

Together, these data demonstrate that rhFVIIa induces dilution-dependent interference in clot-based FV activity assays at low plasma dilutions, which can be effectively mitigated by increased dilution, while TXA does not interfere with aPTT or FV activity measurements.
